# Unveiling Silent Patent Ductus Arteriosus with COVID-19

**DOI:** 10.1590/0037-8682-0086-2025

**Published:** 2025-07-07

**Authors:** Nurhayat Yakut, Kahraman Yakut, Mehmet Gumustas, Serap Bas, Ibrahim Cansaran Tanidir

**Affiliations:** 1 Basaksehir Cam and Sakura City Hospital, Department of Pediatrics, Division of Pediatric Infectious Diseases, Istanbul, Turkey.; 2 Basaksehir Cam and Sakura City Hospital, Department of Pediatrics, Division of Pediatric Cardiology, Istanbul, Turkey.; 3 Basaksehir Cam and Sakura City Hospital, Department of Radiology, Istanbul, Turkey.

A 16-year-old boy with a previous diagnosis of premature silent patent ductus arteriosus (PDA) was referred to the pediatric infectious disease ward due to COVID-19 pneumonia. Lung auscultation revealed bilateral rhonchi and fine crackles in the lower zones. Initial laboratory tests showed that the absolute lymphocyte count was 700 cells/μL, C-reactive protein level 182 mg/dL, procalcitonin level 6.7 ng/mL, prothrombin time/International Normalized Ratio 1.45, and D-dimer 7.5 μgFEU/mL The patient was started on teicoplanin, ceftriaxone, low-molecular-weight heparin, and aspirin as anticoagulant therapy. Echocardiography revealed hemodynamically significant PDA with thrombosis at the origin of the left pulmonary artery. Anticoagulant therapy was continued. Computed tomography angiography revealed focal thrombus formation near the ostium of the pulmonary artery adjacent to the PDA. A marked tortuosity with aneurysmal changes was seen at the PDA level. A partial thrombus was observed in the pulmonary artery supplying the right lower lobe, and cavitary lesions were observed in the lungs (Figure 1A,B,C). The patient’s clinical condition improved, with fever and cough subsiding. Intravenous antibiotics were continued for four weeks. He was discharged in good clinical condition with ongoing anticoagulant treatment. Three months after discharge, his PDA was closed using a transcatheter method. Although potential complications of clinically silent PDA include infective endarteritis and aneurysmal dilation, there is no consensus regarding routine antibiotic prophylaxis and closure^1^ ^-^ ^3^. This case highlights the importance of individualized treatment plans for COVID-19-associated hypercoagulability^4^ and silent PDA, suggesting that early intervention prevents complications.

**FİGURE 1:**
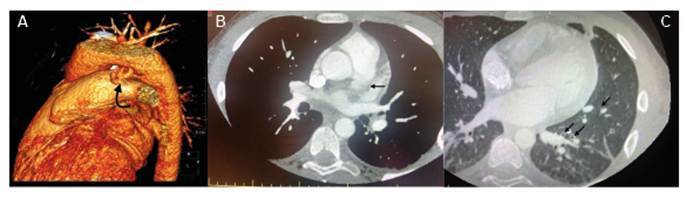
A: 3D image of the patent ductus arteriosus (PDA). B: Partial thrombus in the pulmonary artery. C: Cavitary lesions associated with septic emboli in the lung.

**Ethical Information:** Written informed consent, including the reported images and detailed medical history, was obtained from the patient’s parents for their contributions and permission to publish.

## References

[1] Gillam-Krakauer M, Mahajan K, StatPearls (2023). Patent Ductus Arteriosus.

[2] Bhat YA, Almesned A, Alqwaee A, Al Akhfash A (2021). Catheter Closure of Clinically Silent Patent Ductus Arteriosus Using the Amplatzer Duct Occluder II-Additional Size: A Single-Center Experience. Cureus.

[3] Wu P, Zheng C, Zhang F, Wang P, Zhang H, Chen G (2023). Pulmonary artery aneurysm caused by infective endarteritis attributed to patent ductus arteriosus in children: a case report and literature review. Front Pediatr.

[4] Conway EM, Mackman N, Warren RQ, Wolberg AS, Mosnier LO, Campbell RA (2022). Understanding COVID-19-associated coagulopathy. Nat Rev Immunol.

